# Compatibilization of Cellulose Nanocrystal-Reinforced Natural Rubber Nanocomposite by Modified Natural Rubber

**DOI:** 10.3390/polym16030363

**Published:** 2024-01-29

**Authors:** Punyarat Jantachum, Pranee Phinyocheep

**Affiliations:** Department of Chemistry, Faculty of Science, Mahidol University, Rama VI Rd., Bangkok 10400, Thailand; maypunyarat.j@gmail.com

**Keywords:** cellulose nanocrystals, compatibilizer, modified natural rubber, nanocomposite, mechanical properties

## Abstract

Due to global warming and environmental concerns, developing a fully bio-based nanocomposite is an attractive issue. In this work, the cellulose nanocrystals (CNCs) extracted from *Luffa cylindrica*, a renewable resource, were explored as a bio-based reinforcing filler in natural rubber (NR) nanocomposites. In addition, modified natural rubber was explored as a potential compatibilizer to assist the filler dispersion in the rubber nanocomposite. The effect of the CNC content (0–15 phr) on cure characteristics and the mechanical, dynamic, and thermal properties of NR/CNC nanocomposites was investigated. The results showed that the scorch time and cure time of the nanocomposites increased with increased CNC contents. The optimum tensile strength of NR nanocomposites having 5 phr of the CNC (NR-CNC5) was 20.60% higher than the corresponding unfilled NR vulcanizate, which was related to the increased crosslink density of the rubber nanocomposite. The incorporation of oxidized-degraded NR (ODNR) as a compatibilizer in the NR-CNC5 nanocomposite exhibited a considerably reduced cure time, which will lead to energy conservation during production. Moreover, the cure rate index of NR-CNC5-ODNR is much higher than using a petroleum-based silane coupling agent (Si69) as a compatibilizer in the NR-CNC5 nanocomposite. The good filler dispersion in the NR-CNC5 nanocomposite compatibilized by ODNR is comparable to the use of Si69, evidenced by scanning electron microscopy. There is, therefore, a good potential for the use of modified NR as a bio-based compatibilizer for rubber nanocomposites.

## 1. Introduction

Nowadays, global warming has emerged as a prominent global concern due to excessive greenhouse gases, affecting significant changes in the global climate. Therefore, the utilization of bio-based materials from agricultural waste has great importance in reducing greenhouse gas emissions, offering a possible solution to the issues associated with the global environment and energy resources [1,2]. Cellulose nanocrystals (CNCs) are a natural, sustainable, and high-impact nanomaterial that can be extracted from natural sources such as sisal [3], coconut husk [4], oil palm [5], bagasse [6], bamboo [7], ramie [8], and *Luffa cylindrica* [9]. It has been reported that the outstanding properties of the obtained CNCs, including a large surface-to-volume ratio, high flexibility, high tensile strength, high stiffness, and good thermal properties, play important roles in the research of nanotechnology for the development of novel and advanced materials [10]. Nanocomposites containing CNC as a reinforcing filler have been of great interest in recent years due to their outstanding properties, i.e., renewable in nature, biocompatibility, high tensile strength, stiffness, flexibility, and good electrical and thermal properties. In addition, the extensive surface area of CNC facilitates many interactions between the fiber and matrix; hence, the polarity between fiber and rubber significantly impacts the characteristics of the nanocomposite [11]. Pasquini and coworkers obtained CNCs from cassava bagasse with an aspect ratio of 76 and investigated various CNC contents (0, 2, 5, 7, and 10 wt%) on the dynamic and mechanical properties of NR nanocomposite [12]. They found that the storage modulus and tensile strength of NR nanocomposites increased with increased CNC contents. In contrast, the addition of CNCs results in a reduction in the magnitude of the tan δ peak. Flazino et al. used CNCs with an aspect ratio of 40 to reinforce NR and reported an increase in tensile modulus, yield stress, and tensile strength of NR nanocomposites with increased CNC loading up to 5 wt%; beyond this amount, aggregation of the CNC occurred [13]. This may be due to the effect of the aspect ratio of CNCs on the reinforcing efficacy. In addition to the size of the CNC, the compatibility of the NR matrix and the CNC has to be taken into consideration. The addition of compatibilizers, including coupling agents, or surface functionalization of polar fillers are methods to improve the dispersion of the filler in the rubber matrix, assisting the interaction between the two phases. Roy and Potiyaraj incorporated malleated natural rubber (MNR) (5, 10, and 15 phr) into microcrystalline cellulose (MCC)-reinforced NR composites [14]. They found significant improvement in hardness, modulus, and tensile strength of 5 phr MCC in NR composites compatibilized with 10 phr of MNR compared to the composite without the compatibilizer. The hydroxyl group of MCC could interact with the succinic anhydride groups of MNR, leading to better MCC dispersion in the rubber matrix than the uncompatibilized composite. The increased thermal stability of the NR/MCC composite was also noted when the compatibilizer was included. Somseemee and coworkers modified the surface of cellulose nanofibrils (CNFs) using bis-(triethoxysilyl-propyl) tetrasulfide (TESPT or Si69). They then studied the effect of TESPT-CNF (0–10 phr) to reinforce NR and found that the modulus and hardness of the NR/TESPT-CNF composites increased continuously with increased filler loading [15]. In comparison with untreated CNFs, NR/TESPT-CNFs demonstrated superior mechanical properties at all filler loadings as a result of the enhanced rubber–filler interaction and crosslink density. The highest tensile strength of the NR/TESPT-CNF was obtained at 5 phr filler loading. Jantachum and coworkers reported the incorporation of Si69 in an NBR/NR (50/50) nanocomposite filled with CNCs and found that Si69 facilitated the interaction of CNCs and the polymer matrix, leading to an enhancement in mechanical characteristics in comparison to nanocomposites without Si69 but only at 1 phr in the CNC [16]. Hence, the Si69 might not be appropriate for the NBR/NR nanocomposite filled with CNCs. An alternative compatibilizer has to be taken into account. NR, a bio-polymer, can be modified into various types of functional materials including an increased polarity; hence, it is of interest to use it as a compatibilizer for NR/CNC nanocomposites. 

In this research, NR nanocomposites filled with CNCs extracted from *Luffa cylindrica* were studied. The cure characteristics, mechanical and dynamic properties, thermal stability, and morphology of the NR/CNC nanocomposites filled with different amounts of CNCs were investigated. In addition, oxidized degraded NR (ODNR) was prepared and used as a bio-based compatibilizer for the NR/CNC nanocomposite, compared to the use of a conventional petroleum-based silane coupling agent (Si69).

## 2. Materials and Methods

### 2.1. Materials 

High-ammonia NR latex (60.2% DRC) was a product from Thai Rubber Latex Corporation (Chonburi, Thailand). The CNC was isolated from *Luffa cylindrica* (L.) Rox. (sponge gourd), purchased from Nong Bua Ban community enterprise, Udon Thani, Thailand. The extraction method of the CNC was reported in our previous publication [9]. A total of 35% hydrogen peroxide (H_2_O_2_) was obtained from QRёC Chemical Co., Ltd. (Auckland, New Zealand). Formic acid was a product of Carlo Erba Reagent (Milan, Italy). Sodium nitrite (NaNO_2_) and Tergitol-15-s-15 were supplied by Sigma Aldrich (St. Louis, MI, USA). Methanol, toluene, and dichloromethane were supplied by Labsystems (Samutprakarn, Thailand). Curing agents, i.e., zinc oxide (ZnO), stearic acid, 2,6-di-tert-butyl-p-cresol (BHT), N-tert-butylbenzothiazole-2-sulphenamide (TBBS), and sulfur, were purchased from Siam Chemical Public (Bangkok, Thailand). Bis(3-triethoxysilylpropyl)tetrasulfide (Si69) was provided by Siam Chemical Public (Bangkok, Thailand). 

### 2.2. Preparation of the Pre-Dispersed NR/CNC Nanocomposite

The pre-dispersion of the CNC was prepared by putting 1 g of the CNC in distilled water (20 mL) under stirring at 30 °C for 10 min before the addition of NR latex (167 mL). The dispersed CNC in NR latex was stirred at 30 °C for 30 min. The contents of CNCs varied from 0 to 15 phr. Finally, the obtained pre-dispersed NR/CNC latex was poured on an aluminum tray and dried at 30 °C until constant weight.

### 2.3. Preparation of Oxidized Degraded NR (ODNR)

A total of 20% DRC of NR latex (300 mL) was stabilized with 5 phr of Tergitol surfactant (3 g) at 30 °C in a glass reactor for 24 h. Then, 5% of formic acid was added to NR latex until pH 5. The latex was heated to 70 °C, and then H_2_O_2_ (5.27 mL) and NaNO_2_ (12.14 g) were simultaneously added. The latex was stirred for 24 h and was then subjected to coagulation in methanol. ODNR was obtained and was then washed with distilled water until neutral pH. It was further purified by re-dissolving in toluene and coagulating in methanol, before drying in a vacuum oven at 60 °C until constant weight. 

### 2.4. Preparation of Rubber Compounding

The compound formulation of NR/CNC nanocomposites is shown in Table 1. Each compound contains the same curing agent, including ZnO (5 phr), stearic acid (2 phr), BHT (1 phr), TBBS (1.5 phr), and sulfur (1.5 phr). The mixing steps are summarized as follows. The pre-dispersed NR/CNC nanocomposites (ODNR or Si69 if needed) were firstly masticated for 5 min on a two-roll mill at room temperature. Next, ZnO, stearic acid, and BHT were filled and mixed for 2 min. Thereafter, the curatives, which were TBBS and sulfur, were put in and mixed for another 5 min. The total mixing time for each compound was kept constant for 12 min. At the end of mixing, 10 end-roll passes were performed in the same direction before sheeting the compound off. NR compounds filled with CNCs are denoted as NR-CNCs and varied contents of CNCs (0, 2, 5, 10, and 15 phr). NR-CNCs filled with ODNR or Si69 are denoted as the NR-CNC5-ODNR and NR-CNC5-Si69, respectively.

### 2.5. Preparation of NR/CNC Vulcanizates

A rubber sheet (a thickness of 1 mm) was compressed at 150 °C and curing periods were obtained from a moving die rheometer (MDR) in Section 2.7 and a force of 10 MPa in a hot press. The obtained vulcanizate was stored for 24 h at room temperature before its properties were determined.

### 2.6. Characterization of ODNR

The FT-IR spectrum of ODNR was recorded using FT-IR (Perkin Elmer model Frontier, Waltham, MA, USA). The sample was dissolved in dichloromethane (CH_2_Cl_2_) and cast on a NaCl cell. The sample was scanned 16 times with 4 cm^−1^ resolutions in transmission mode ranging from 4000 to 400 cm^−1^. 

The ^1^H-NMR spectrum of ODNR was performed on a 500 MHz (5000 scans) Bruker AM 400 spectrophotometer (Bruker Corpora, Karlsruhe, Germany) using about 10 mg of the sample dissolved in deuterated chloroform containing tetramethylsilane as an internal reference. 

The weight-average (M_w_) and number-average molecular weight (Mn) of ODNR were measured by gel permeation chromatography (GPC) (150-C ALC/GPC, Waters, Milford, MA, USA). About 10 mg of ODNRs were dissolved in 10 mL of tetrahydrofuran (THF) and then filtered with a 0.45 μm nylon filter before analysis. The flow rate of the THF eluent was 1 mL/min at 30 °C using a guard column, including an RI detector and polystyrene standard. 

Thermal properties of ODNR and NR/CNC nanocomposites were investigated using a thermogravimetric analyzer (TGA/DTA, Mettler, Toledo, Waltham, MA, USA). About 8–10 mg of the sample was heated from 40 to 600 °C at a heating rate of 20 °C/min under a nitrogen environment. Then, the sample was further heated at the same heating rate until 700 °C under an oxygen atmosphere.

### 2.7. Characterization of Rubber Nanocomposites

Cure characteristics of rubber compounds, i.e., minimum torque (M_L_), maximum torque (M_H_), delta torque (M_H_-M_L_), scorch time (t_s2_), cure time (t_c90_), and cure rate index (CRI), were determined by moving the die rheometer (MDR, MonTech, MDR 3000 M, Baden-Württemberg, Germany) at 150 °C.

The crosslink density of each rubber vulcanizate was determined using Equations (1) and (2), according to Flory–Rhener theory [17]. The rubber vulcanizate specimen with a 1 mm thickness was cut into a square shape of about 1 × 1 cm^2^ and then weighed before being immersed in toluene (50 mL) for 7 days. The swollen sample was removed from toluene, and the excess toluene was blotted with a paper towel before the weight was measured.

(1) $$-\left[\ln\left(1-\phi_{r}\right)+\phi_{r}+\chi \phi_{r}^{2}\right]=V_{0}n\left[\phi_{r}^{\frac{1}{3}}-\frac{\phi_{r}}{2}\right]$$

(2) $$\frac{1}{\phi_{r}}=1+\left[\frac{w_{2}}{w_{1}}\times \frac{x\rho_{r}}{x\rho_{s}}\right]$$

where *ϕ_r_* is the volume fraction of rubber in the swollen mass, *χ* is the interaction coefficient between the rubber network and solvent (0.3795), *V*_0_ is the molar volume of the solvent (106.2), *n* is the physical degree of crosslinking, and *ρ_r_* and *ρ_s_* are the densities of the rubber (NR = 0.92 mol/cm^3^) and solvent (toluene = 0.87 mol/cm^3^), respectively. 

The hardness of rubber vulcanizates was determined by a hardness tester (Hardness (Shore A), H17A, Wallace, Surrey, England) following ASTM D2240-97 [18]. The specimen with a 6 mm thickness was used, and an average of 6 positions was reported as the hardness value.

Tensile properties of rubber vulcanizates were measured under ASTM D412-98 [19] using an Instron 5566 (High Wycombe, UK). The tensile dumbbell-shaped specimens were prepared from the vulcanized sheets using a type C die. The crosshead speed of 500 mm/min using a full-scale force of 1 kN was carried out. 

A dynamic mechanical analyzer (DMA) (DMTA model GABO, EPLEXOR QC 25, Bavaria, Germany) was used to determine the dynamic properties of the nanocomposites. A specimen with dimensions of 30 × 10 × 2 mm^3^ was used for the measurement. The experiments were carried out with the pre-strained specimen at 1% and 10 Hz of 0.1% dynamic strain using a temperature ranging from −80 °C to 100 °C at a heating rate of 2 °C/min and a strain amplitude of 0.01%. 

The morphology of rubber vulcanizates was examined using a field-emission scanning electron microscope (FE-SEM, model SU-8010, Hitachi, Ibaraki, Japan). The fracture surface of rubber vulcanizates in liquid N_2_ was coated with platinum to prevent the electron bombard on the sample surface using an accelerating voltage of 10 kV.

## 3. Results and Discussion

### 3.1. Analysis of ODNR

**Figure 1:**
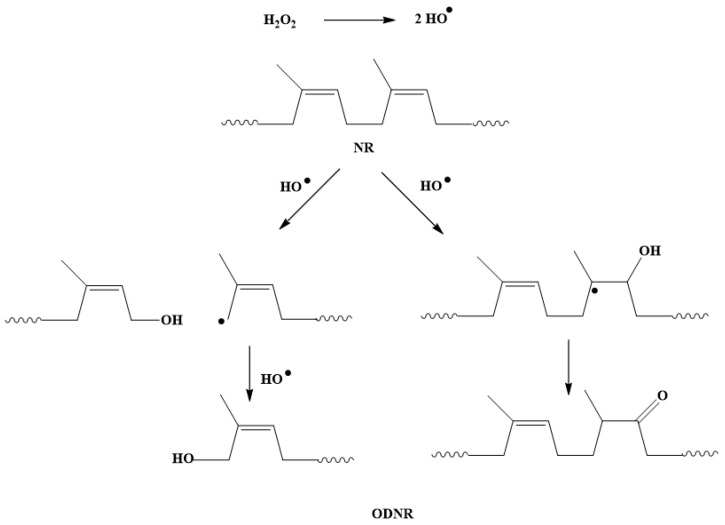
Proposed oxidative degradation of NR to ODNR.

NR, a bio-based polymer consisting of unsaturated repeating units, has the potential for various chemical modifications. It can be transformed from a non-polar character into a material having polar functionality to make it a high-value-added material that can be further used as a compatibilizer for filled polymer composites or polymer blends. In this work, the modification of NR was carried out using H_2_O_2_ for oxidative degradation. The isoprene repeating unit of NR is prone to the reaction at the C=C and the reaction at the carbon next to the double bond. The H_2_O_2_ was dissociated into hydroxyl radicals, which react at isoprene units of NR, leading to chain scission and the formation of hydroxyl and carbonyl functional groups, as proposed in Figure 1. These functional groups have the potential to react with the hydroxyl groups of CNCs in the NR/CNC nanocomposites.

**Figure 2:**
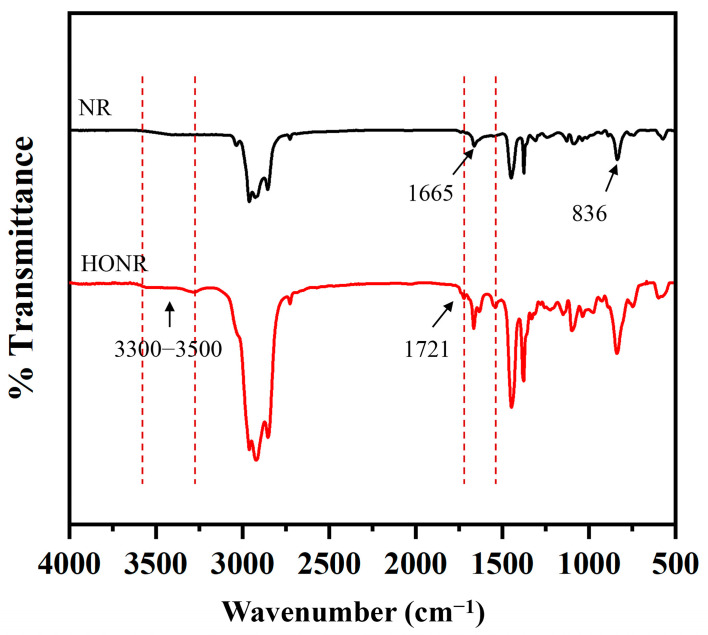
FT-IR spectra of NR and ODNR.

FT-IR spectra of NR and ODNR in Figure 2 reveal the peaks at 836 and 1665 cm^−1^, attributed to =CH wagging and stretching vibration of the C=C bond, respectively, which are commonly found as the fingerprint region of NR. After oxidative degradation, the FT-IR spectrum of ODNR displayed two more peaks compared to NR at 1721 and 3300 cm^−1^, and were assigned to the carbonyl and hydroxyl groups, respectively, similar to previous reports [20,21].

**Figure 3:**
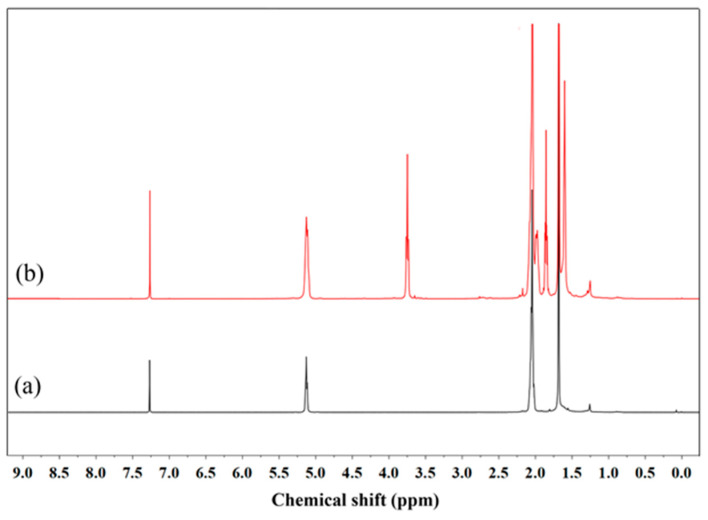
^1^H-NMR spectra of (**a**) NR and (**b**) ODNR.

Figure 3a shows chemical shifts of proton signals of NR at 1.65, 2.05, and 5.10 ppm, assigned to –CH_3_, –CH_2_-C=C, and methine proton, respectively. Additionally, the spectrum of ODNR included peaks that were comparable to NR, with an extra small peak at 3.77 ppm, which represented the methylene protons of –CH_2_OH and occurred from the oxidative degradation of NR [21].

GPC was applied to determine the molecular weight of modified NR and NR. Physically, the obtained ODNR was a yellowish, sticky, and viscous material, indicating the scission of the NR chain. The M_n_ of ODNR was reduced from 908,000 g/mol (M_n_ of NR) to 83,400 g/mol. Additionally, the M_w_ of ODNR was also reduced from 1,200,000 g/mol (M_w_ of NR) to 384,400 g/mol. These results follow previous reports that H_2_O_2_ causes hydrocarbon chain degradation [21,22]. Moreover, the polydispersity index (PDI) of ODNR is 4.1, which increased compared to NR (1.3) because random chain scission occurred.

**Figure 4:**
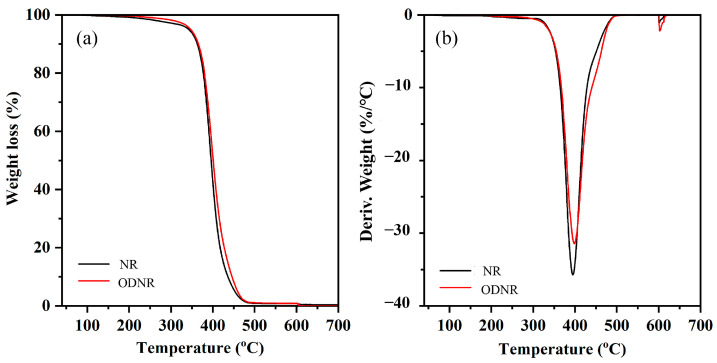
(**a**) TGA and (**b**) DTG of NR and ODNR.

The thermal stability of ODNR was examined using TGA, which is a technique used to evaluate the mass change in a sample as a function of temperature. TGA and the derivative of TGA (DTG) curves of NR compared to ODNR are shown in Figure 4. It was found that the TGA and DTG curves of NR and ODNR exhibit one main step of weight loss and a single decomposition temperature (T_d_). The T_d_ of NR was observed at 394 °C, which is attributed to the isoprene unit decomposition [16]. Furthermore, ODNR shows a higher T_d_ than NR, which was observed at 399 °C. This is attributed to the hydrogen bonding of the hydroxyl and carbonyl groups in ODNR [21]. Additionally, intermolecular interactions of ODNR affect the high heat resistance of modified rubber, resulting in a direct link between the bond energies in the polymer structure [23,24].

### 3.2. Cure Characteristics of NR/CNC Nanocomposites

**Figure 5:**
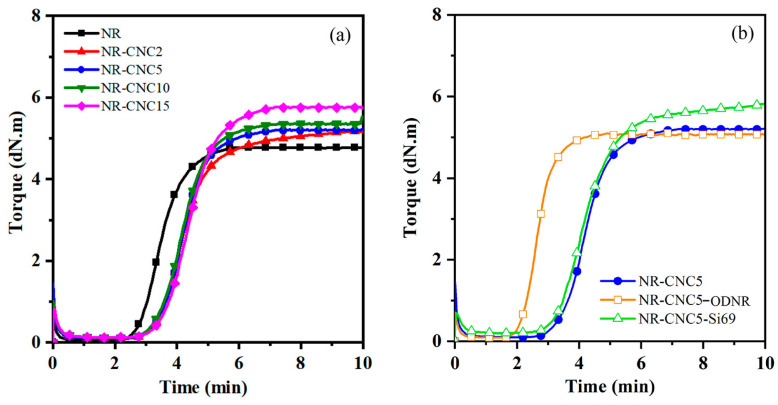
(**a**) NR/CNC nanocomposites at various contents of CNCs and (**b**) NR-CNC5 with ODNR and Si69 nanocomposites.

The cure curves of NR/CNC nanocomposites with different CNC contents are depicted in Figure 5a. Table 2 represents the cure properties of NR/CNC nanocomposites, including M_H_, M_L_, t_s2_, t_c90_, and CRI. The M_H_ of NR/CNC nanocomposites increased with increased CNC contents. The increased stiffness of NR/CNC nanocomposites limited the mobility of polymer chains [14,25]. Additionally, the t_s2_ and t_c90_ of all nanocomposites tend to increase with increasing CNC contents. This may occur as a result of an increase in the hydroxyl group content of CNCs that absorb accelerators and curing agents of the rubber compounds, retarding the curing reaction [16,17]. Figure 5b shows cure curves of NR-CNC5 with ODNR and Si69 nanocomposites. It can be seen that the cure curve of the NR-CNC5-ODR was shifted to a lower reaction time. The results in Table 2 show that the t_s2_ and t_c90_ of NR-CNC5-ODNR nanocomposites were the lowest compared to NR, NR-CNC, and NR-CNC5-Si69 nanocomposites. The t_c90_ referred to the vulcanization time of the rubber nanocomposites. The t_c90_ of NR-CNC5-ODR is 3.24 min, which is faster than NR-CNC5 (5.42 min) and NR-CNC5-Si69 (5.44 min), indicating about 40% improvement in curing time. The functional groups (hydroxyl and carbonyl groups) of ODNR might have interacted with the hydroxyl groups of the CNC, inhibiting the CNC from absorbing the curing agents [10]. In addition, the hydroxyl groups of ODNR could form hydrogen bonds toward the carbonyl functions of ODNR and accelerate the vulcanization process [17]. This result implies that using ODNR could save energy in the production process. In addition, the CRI of NR-CNC5-ODNR is significantly greater than NR-CNC5-Si69, suggesting that the nanocomposite with an ODNR compatibilizer was facile to process and better than the use of a petroleum-based coupling agent (Si69).

### 3.3. Crosslink Density of NR/CNC Nanocomposites

**Figure 6:**
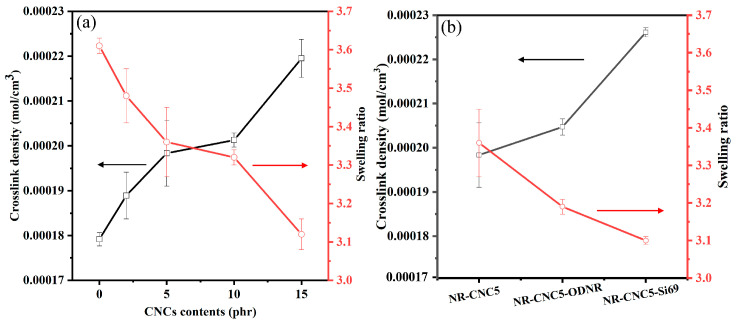
Crosslink density and swelling ratio of (**a**) NR/CNC nanocomposites at various contents of CNCs and (**b**) NR-CNC5 with ODNR and Si69 nanocomposites.

The crosslink density and swelling ratio of NR/CNC nanocomposites having various contents of CNCs are displayed in Figure 6. It is seen in Figure 6a that the crosslink density of all nanocomposites tends to increase with an increase in CNC contents, while the swelling ratio tends to decrease. The explanation can be due to the higher filler–rubber interaction of nanocomposites related to the increased maximum torque of the nanocomposites [16]. The swelling ratio and crosslink density of NR-CNC5 with ODNR and Si69 nanocomposites are shown in Figure 6b. The lower swelling ratio is in accordance with the higher crosslink density of NR-CNC5-ODNR and NR-CNC5-Si69 compared with NR-CNC5. This might be due to the higher filler–rubber interactions that affect the segmental mobility of the rubber chains. The higher crosslink density implied the higher formation of the rubber network, leading to the improved mechanical properties of nanocomposites [26,27]. 

### 3.4. Mechanical Properties 

**Figure 7:**
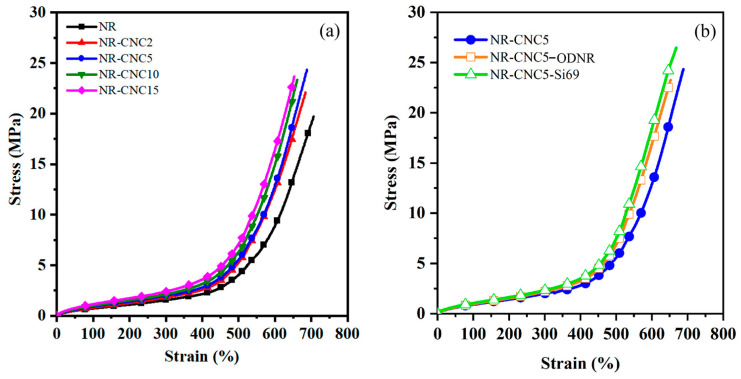
Stress–strain curves of (**a**) NR/CNC nanocomposites at various CNC contents and (**b**) NR-CNC5 with ODNR and Si69 nanocomposites.

Figure 7a demonstrates stress–strain curves of NR/CNC nanocomposites at various contents of CNCs. The stress of all nanocomposites tends to shift to low strain with increasing the CNC content. The explanation can be due to the addition of a rigid filler in the composite, which increases the modulus, or the detachment of the filler from the rubber matrix [27,28], relating to the decreasing storage modulus of the composites. In addition, the tensile strength of nanocomposites was found to be improved when the CNC was added to the NR nanocomposite. Comparing the samples with unfilled NR vulcanizate, the tensile strength increased by 19.75%, 20.60%, 17.49%, and 19.70% for NR-CNC composites containing 2, 5, 10, and 15 phr of CNCs, respectively. It could be noted that the CNC has a high aspect ratio and specific surface area and is expected to have an increased fiber matrix contact area, resulting in a good load transfer behavior [29]. The prepared CNCs used in this work contain negative charges on CNC surfaces, leading to the reduced aggregation of CNC suspensions [9]. Additionally, the increase in tensile strength of NR-CNC nanocomposites might be a result of the increase in crosslink density caused by the filler–rubber linkages [30], which is shown in Figure 6. Consequently, this can provide the high reinforcing capability of CNCs in the rubber nanocomposites [31]. Moreover, the tensile strength of NR/CNC nanocomposites with 5 phr of CNC contents showed the highest value. This may be due to the better dispersion of CNCs in NR nanocomposites than other nanocomposites, which can be confirmed by the SEM image, which will be discussed later. The improvement of interactions between the CNC and the polymer matrix via compatibilizers was considered. NR was transformed into ODNR with hydroxyl and carbonyl functional groups that might form a linkage between the hydroxyls of CNCs and the non-polar nature of the NR main chains [32]. ODNR material might be a potential compatibilizer for the NR/CNC composites; however, it is required to compare with the commercially available Si69. Therefore, NR-CNC5-ODNR and NR-CNC5-Si69 composites were prepared. Figure 7b reveals that the tensile strength of NR-CNC5-ODNR and NR-CNC5-Si69 were higher than NR-CNC5. This might be because of the low molecular weight of ODNR, which gives ODNR greater ability to flow and a greater tackiness, resulting in a greater adhesion of the filler and rubber matrix [33,34]. NR, a bio-based polymer consisting of unsaturated repeating units, has the potential for various chemical modifications. It can be transformed from a non-polar character into a material with polar functionality, which makes it a high-value-added material that can be further used as a compatibilizer for filled polymer composites or polymer blends. In this work, the modification of NR was carried out using H_2_O_2_ for oxidative degradation. The isoprene repeating unit of NR is prone to the reaction at C=C and the reaction at the carbon next to the double bond. The H_2_O_2_ was dissociated into hydroxyl radicals, which react at isoprene units of NR, leading to chain scission and the formation of hydroxyl and carbonyl functional groups, as proposed in Figure 1. These functional groups have the potential to react with the hydroxyl groups of CNCs in the NR/CNC nanocomposites.

The elongation at the break tended to decrease when CNC contents increased. The decreased chain mobility led to a reduction in elasticity [35,36]. At a high strain in the NR/CNC composites, the tensile stress abruptly rises due to the crystallization of NR before the tensile specimen ruptures. The upturned point of 2, 5, 10, and 15 phr of CNC contents in NR composites in Figure 7a is observed at about 530%, 520%, 500%, and 490% strains, respectively. The results infer that strain-induced crystallization occurs at the lower strain compared with unfilled NR vulcanizate (560%). High elongation and tensile stress at the break are related to the high crosslink density of the nanocomposites. It can be concluded that the CNC was effective in reinforcing the NR nanocomposite. Additionally, the elongation at the break of NR-CNC5-ODNR (656%) and NR-CNC5-Si69 (669%) slightly shifts to lower strains compared to NR-CNC5 (677%). Moreover, the upturn in stress of NR-CNC5-ODNR and NR-CNC5-Si69 nanocomposites were 480 and 460%, respectively, which can be attributed to strain-induced NR crystallization [37]. The interfacial adhesion of the CNC and NR matrix may be enhanced after the addition of a compatibilizer and coupling agent [14,17]. As a result, ODNR might act as a good bio-based compatibilizer, similar to the commercially available silane coupling agent, in enhanced mechanical properties.

### 3.5. Dynamic Mechanical Properties 

**Figure 8:**
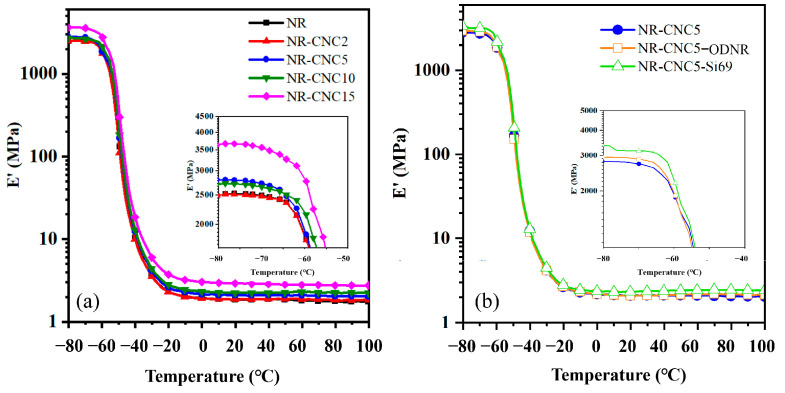
Plots of the storage modulus (E′) versus temperatures of (**a**) NR/CNC nanocomposites at various CNC contents and (**b**) NR-CNC5 with ODNR and Si69 nanocomposites.

DMA was used to examine the compatibility and interaction of NR and CNCs in the nanocomposites. Figure 8 shows the storage modulus (E′) of NR/CNC nanocomposites at various CNC contents without and with ODNR and Si69. The filler network or filler–filler interaction is the primary contributor to the storage modulus below T_g_ due to the restriction of particulate motion in the polymer matrix [38]. Figure 8a reveals that the E′ of the nanocomposites tends to increase with increased CNC contents because the movement of polymer chains is reduced due to the obstacle by CNCs in the rubber matrix [17]. For the range of −65 °C to −10 °C, the E′ of all nanocomposites decreased when the temperature increased, as the mobility of rubber chains augmented [38]. In addition, the E′ of the nanocomposite containing higher CNC contents was higher than the nanocomposite containing lower CNC contents. This is because the CNC can transfer stress to the rubber matrix, inducing an increase in the E′ of the composites [17,39]. When the temperature increases to ambient temperature or above, the movement of the rubber chains can occur, resulting in the filler–rubber interaction and the rubber network structure becoming dominant in the E′ [40]. The E′ of the nanocomposites increases with increased CNC contents. This may be the result of filler–rubber linkages causing a greater density of crosslinks, and the increased torque differences provide further evidence. In the case of NR-CNC5-ODNR and NR-CNC5-Si69 (Figure 8b), it can be seen that below −65 °C_,_ the nanocomposites with the compatibilizer showed higher E′ than NR-CNC5 nanocomposites. This may be due to a stronger filler–rubber network in nanocomposites with the compatibilizer compared to the NR-CNC5 nanocomposites without the compatibilizer [41,42]. When the temperature increases to ambient temperature or above, the mobility of the rubber chains increased, and filler–rubber interaction and the rubber network structure become dominant in E′ [39]. From the results, it can be suggested that the nanocomposites with ODNR and Si69 have a stronger filler–rubber interaction than the NR-CNC5 nanocomposite without compatibilizers. Moreover, the better CNC dispersion in the rubber matrix resulted from the addition of the ODNR compatibilizer and a silane coupling agent [16,17].

**Figure 9:**
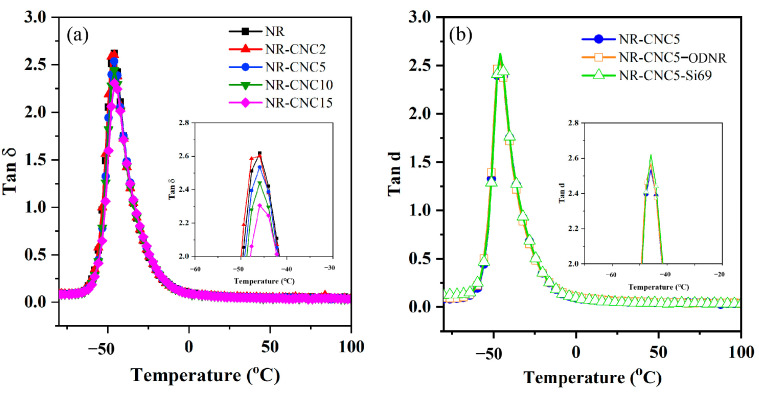
Temperature dependence of loss factor tan δ of (**a**) NR/CNC nanocomposites at various contents of CNC and (**b**) NR-CNC5 with ODNR and Si69 nanocomposites.

The tan δ of NR/CNC nanocomposites was investigated, as displayed in Figure 9a. All nanocomposites showed only one peak height of the rubber, which can be used to evaluate the glass transition temperature (T_g_) and determine the reinforcement of the rubber composite. The T_g_ values of the nanocomposites slightly shifted toward higher temperatures when the CNC content increased, compared to unfilled NR vulcanizate. This might be due to the increase in filler–rubber interaction or stiffness of the nanocomposites. Thus, a higher temperature was required for the movement of rubber chains [43,44]. Moreover, the tan δ_max_ of nanocomposites decreases with increasing CNC contents due to the restraining of the polymer movement and filler–rubber interaction [17]. The tan δ_max_ of NR-CNC5-ODNR and NR-CNC5-Si69 compared with NR-CNC5 is investigated as displayed in Figure 9b. The results showed that NR-CNC5-ODNR and NR-CNC5-Si69 nanocomposites showed a slightly higher tan δ_max_ than NR-CNC5 nanocomposites. These results indicate that less rubber was trapped in the CNC network, and there were still more flexible rubber chains to respond to the dynamic deformation [45].

### 3.6. Morphology of NR/CNC Nanocomposites

**Figure 10:**
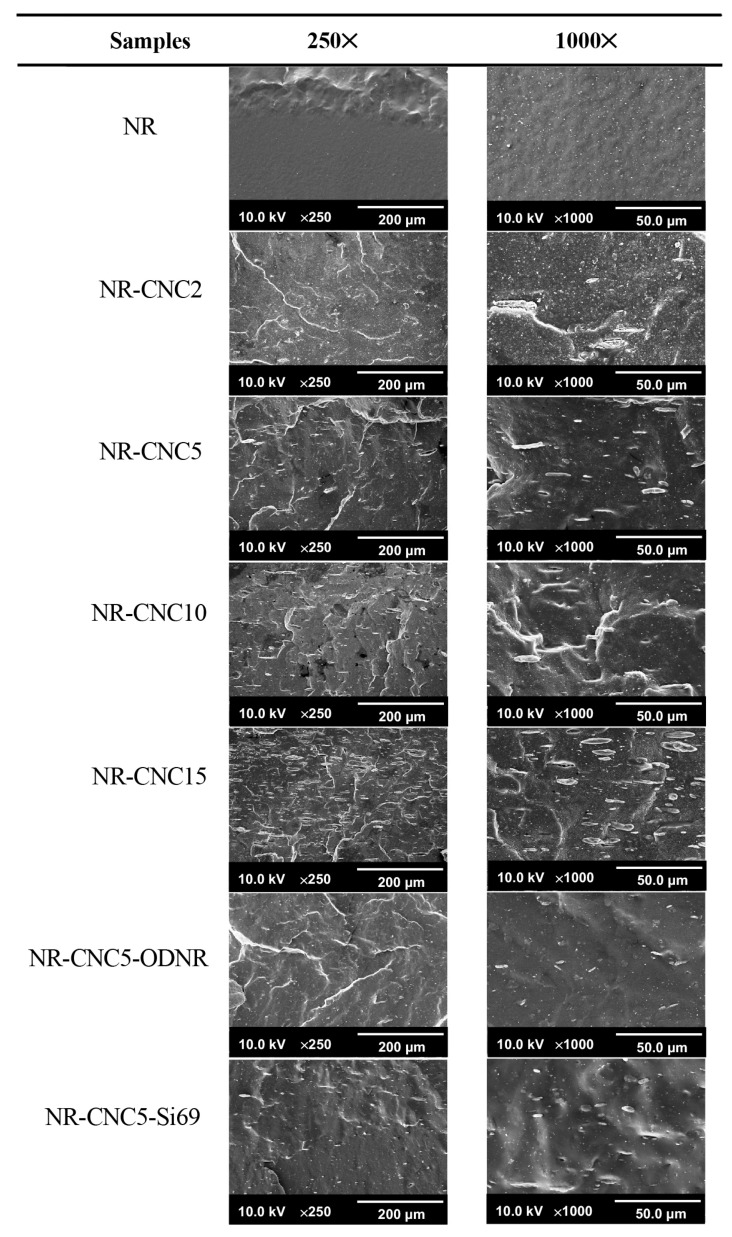
FE-SEM micrographs of NR/CNC nanocomposites at various CNC contents and NR-CNC5 with ODNR and Si69 nanocomposites at low and high magnifications.

The morphology of NR/CNC nanocomposites at various CNC contents was investigated using the FE-SEM technique, as shown in Figure 10. It was discovered that the fractured surface of unfilled NR vulcanizate was smooth. When incorporating the CNC into the nanocomposite, a rough surface was found, and increased CNC contents revealed an uneven surface and agglomeration of CNCs, including some voids between the CNC and rubber matrix due to the poor adhesion at their interface. Another reason might be due to the nature of the CNC, in which hydrogen bonding occurs between the hydroxyl groups of the filler forming aggregation after drying [14,46]. Nevertheless, most of the NR/CNC nanocomposites showed homogeneous filler dispersion in the NR matrix, which is the key factor for improved tensile strength of the NR/CNC nanocomposites shown in the previous results. In addition, the smoother surface and good dispersion of CNCs in the NR matrix were observed in NR-CNC5-ODNR and NR-CNC5-Si69 compared to NR-CNC5 nanocomposites, demonstrating increased interfacial compatibility between the filler and the rubber matrix [47]. As a result, ODNR can improve the filler–rubber interaction in the nanocomposite and is comparable to the use of Si69.

### 3.7. Thermal Stability of NR/CNC Nanocomposites

**Figure 11:**
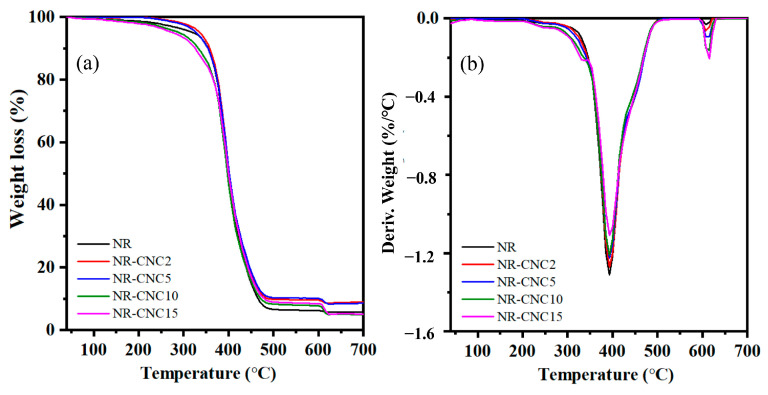
(**a**) TGA and (**b**) DTG curves of NR/CNC nanocomposites at various contents of CNCs.

**Figure 12:**
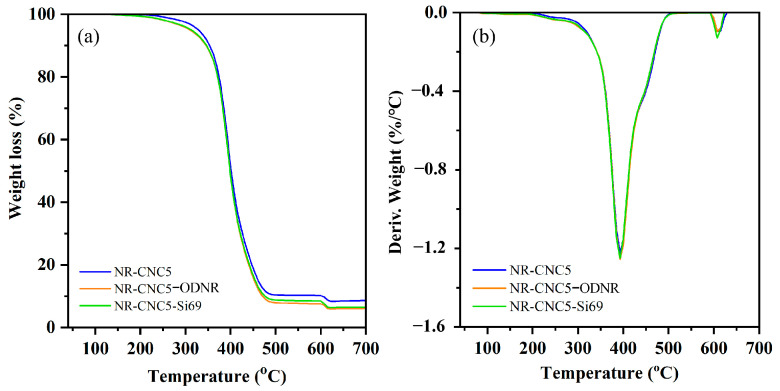
(**a**) TGA and (**b**) DTG curves of NR-CNC5 with ODNR and Si69 nanocomposites.

The TGA and DTG of NR/CNC nanocomposites were performed, as illustrated in Figure 11a and Figure 11b, respectively. Figure 10 reveals the initial mass loss at the temperature of 100 °C, which is due to the combustion of organic additives applied for vulcanization and the loss of adsorbed moisture from the CNCs. Subsequently, the values of temperature at 5% mass loss (T_5%_) and the decomposition temperature at maximum weight loss (T_max_) are shown in Table 3. Loss of final mass under O_2_ occurs between 600 and 700 °C and is related to the residual char formation of the CNC [48]. The T_5%_ and T_max_ at 195 °C and 392 °C indicate NR decomposition, respectively [1,40]. It was found that the T_5%_ of the NR-CNC2 (203 °C) and NR-CNC5 (202 °C) nanocomposites were slightly higher than other nanocomposites and unfilled NR, with a slight increase in the T_max_ of the NR-CNC5 (393 °C) nanocomposite compared to the unfilled NR composite (392 °C). The decomposition temperature of nanocomposites containing 10 and 15 phr of CNCs was lower than NR nanocomposites containing 2 and 5 phr of CNCs. This may be the result of CNCs having comparatively lower thermal stability than NR [1,15], for which CNCs showed T_max_ at 336 °C in our previous work [9]. Figure 12a presents TGA curves of the NR-CNC5-ODNR and NR-CNC5-Si69 compared to NR-CNC5 nanocomposites. The degradation profile and mass loss of the nanocomposites with ODNR and Si69 are similar to the TGA curves of nanocomposites without compatibilizers. The result showed that the decomposition temperatures of NR-CNC5-ODNR and NR-CNC5-Si69 are not different in T_max_ compared with NR-CNC5 (393 °C). The amount of the CNC may be too low, which did not influence the T_max_ of the nanocomposites. The T_5%_ of NR-CNC5-ODNR and NR-CNC5-Si69 is about 205 °C and 207 °C, respectively, which is higher than NR-CNC5 (202 °C). Therefore, the addition of ODNR and Si69 compatibilizers can improve T_5%_ of nanocomposites.

## 4. Conclusions

In this research, NR nanocomposites filled with cellulose nanocrystals (CNCs) extracted from *Luffa cylindrica*, a renewable resource, were successfully carried out. The CNC was then incorporated in NR to prepare NR/CNC nanocomposites. Given the polarity difference between the CNC and NR, a compatibilizer might be needed to enhance the compatibility between the two phases. Therefore, a compatibilizer derived from NR was taken into consideration. The ODNR compatibilizer was successfully prepared by an oxidative degradation reaction of NR to transform it into a functional rubber-containing polar functional group. The role of ODNR as a compatibilizer was compared with a commercial coupling agent (Si69). As a result, the curing properties of NR/CNC nanocomposites without a compatibilizer increased with increasing CNC contents. Moreover, the curing time of NR/CNC5-ODNR composites (3.24 min) that contained ODNR as a compatibilizer was shorter than all other NR/CNC nanocomposites and the NR-CNC5-Si69 composite using a petroleum-based silane coupling agent (Si69) (5.44 min). This might be because the interfacial interaction between the CNC and NR matrix was improved by ODNR. The percentage regarding the improvement in cure time observed with NR/CNC5-ODNR compared to NR/CNC5 and NR-CNC5-Si69 is about 40%. The tensile properties and thermal stability of NR nanocomposites were improved most effectively in the presence of 5 phr of CNC contents, in particular with the presence of ODNR. Also, the NR/CNC nanocomposite with ODNR showed an increase in maximum torque and modulus at low strain because the cellulose nanocrystals were spread out well in NR, as evidenced by SEM analysis. Consequently, the mechanical and dynamic properties of the NR-CNC5-ODNR nanocomposite are comparable to adding Si69. As a result, the use of modified NR (ODNR) minimized the curing time, which is faster than the use of a petroleum-derived silane coupling agent, leading to a reduction in processing costs. The fully bio-based nanocomposite of NR/CNC/ODNR is considered a potential material with low CO_2_ emissions.

## Figures and Tables

**Table 1:** Compound formulation of NR/CNC nanocomposites.

Ingredients	Amount (phr)						
	NR	NR-CNC2	NR-CNC5	NR-CNC10	NR-CNC15	NR-CNC5-ODNR	NR-CNC5-Si69
NR	100	100	100	100	100	100	100
CNCs	0	2	5	10	15	5	5
ODNR	-	-	-	-	-	5	-
Si69	-	-	-	-	-	-	0.25

**Table 2:** Cure characteristics of NR/CNC nanocomposites.

Properties	NR	NR-CNC2	NR-CNC5	NR-CNC10	NR-CNC15	NR-CNC5-ODNR	NR-CNC5-Si69
M_H_ (dNm)	4.51	5.10	5.35	5.46	6.99	5.71	6.08
M_L_ (dNm)	0.06	0.10	0.10	0.12	0.13	0.09	0.19
M_H_-M_L_ (dNm)	4.45	5.00	5.25	5.34	6.86	5.62	5.89
t_s2_ (min)	2.80	3.16	3.25	3.32	3.62	2.05	3.36
t_c90_ (min)	3.87	5.37	5.42	5.51	5.56	3.24	5.44
CRI (min^−1^)	93.45	45.24	46.08	45.66	51.54	84.03	48.08

**Table 3:** Thermal properties of NR/CNC nanocomposites.

Samples	T_5%_ (°C)	T_max_ (°C)
NR	195	392
NR-CNC2	203	393
NR-CNC5	202	393
NR-CNC10	199	390
NR-CNC15	199	390
NR-CNC5-ODNR	205	393
NR-CNC5-Si69	207	393

## Data Availability

The data presented in this research are available upon request from the corresponding author.

## References

[1] Koeipudsa N., Chanthateyanonth R., Daniel P., Phinyocheep P. (2022). Development of natural rubber nanocomposites reinforced with cellulose nanocrystal isolated from oil palm biomass. J. Polym. Res..

[2] Lozada E.R., Gutiérrez A.C.M., Jaramillo C.J.A., Sánchez J.C., Barrera Torres G. (2023). Vegetable cellulose fibers in natural rubber composites. Polymers.

[3] Morán J.I., Alvarez V.A., Cyras V.P., Vázquez A. (2007). Extraction of cellulose and preparation of nanocellulose from sisal fibers. Cellulose.

[4] Rosa M.F., Medeiros E.S., Malmonge J.A., Gregorski K.S., Wood D.F., Mattoso L.H.C. (2010). Cellulose nanowhiskers from coconut husk fibers: Effect of preparation conditions on their thermal and morphological behavior. Carbohydr. Polym..

[5] Haafiz M.K.M., Eichhorn S.J., Hassan A., Jawaid M. (2013). Isolation and characterization of microcrystalline cellulose from oil palm biomass residue. Carbohydr. Polym..

[6] Kumar A., Negi Y.S., Choudhary V., Bhardwaj N.K. (2014). Characterization of cellulose nanocrystals produced by acid-hydrolysis from sugarcane bagasse as agro-waste. J. Mater. Phys. Chem..

[7] Hu Y., Tang L., Lu Q., Wang S., Chen X., Huang B. (2014). Preparation of cellulose nanocrystals and carboxylated cellulose nanocrystals from borer powder of bamboo. Cellulose.

[8] Syafri E., Kasim A., Abral H., Asben A. (2018). Cellulose nanofibers isolation and characterization from ramie using a chemical-ultrasonic treatment. J. Nat. Fibers.

[9] Jantachum P., Phinyocheep P. (2023). A simple method for extraction of cellulose nanocrystals from green *Luffa cylindrica* biomaterial and their characteristics. Polym. Int..

[10] Ng H.M., Sin L.T., Tee T.T., Bee S.-T., Hui D., Low C.Y. (2015). Extraction of cellulose nanocrystals from plant sources for application as reinforcing agent in polymers. Compos. Part B.

[11] Fallahi H., Kaynan O., Asadi A. (2023). Insights into the effect of fiber–matrix interphase physiochemical- mechanical properties on delamination resistance and fracture toughness of hybrid composites. Compos. Part A Appl. Sci. Manuf..

[12] Pasquini D., de Morais Teixeira E., da Silva Curvelo A.A., Belgacem M.N., Dufresne A. (2010). Extraction of cellulose whiskers from cassava bagasse and their applications as reinforcing agent in natural rubber. Ind. Crops Prod..

[13] Neto W.P.F., Mariano M., da Silva I.S.V., Silverio H.A., Putaux J.L., Otaguro H. (2016). Mechanical properties of natural rubber nanocomposites reinforced with high aspect ratio cellulose nanocrystals isolated from soy hulls. Carbohydr. Polym..

[14] Roy K., Potiyaraj P. (2017). Development of high performance microcrystalline cellulose based natural rubber composites using maleated natural rubber as compatibilizer. Cellulose.

[15] Somseemee O., Sae-Oui P., Siriwong C. (2021). Reinforcement of surface-modified cellulose nanofibrils extracted from Napier grass stem in natural rubber composites. Ind. Crops Prod..

[16] Jantachum P., Khumpaitool B., Utara S. (2023). Effect of silane coupling agent and cellulose nanocrystals loading on the properties of acrylonitrile butadiene rubber/natural rubber nanocomposites. Ind. Crops Prod..

[17] Moonart U., Utara S. (2019). Effect of surface treatments and filler loading on the properties of hemp fiber/natural rubber composites. Cellulose.

[18] (2015). Standard Test Method for Rubber Property—Durometer Hardness.

[19] (2002). Standard Test Methods for Vulcanized Rubber and Thermoplastic Rubbers and Thermoplastic Elastomers-Tension.

[20] Phinyocheep P., Phetphaisit C.W., Derouet D., Campistron I., Brosse J.C. (2005). Chemical degradation of epoxidized natural rubber using periodic acid: Preparation of epoxidized liquid natural rubber. J. Appl. Polym. Sci..

[21] Ibrahim S., Daik R., Abdullah I. (2014). Functionalization of Liquid Natural Rubber via Oxidative Degradation of Natural Rubber. Polymers.

[22] Suhawati I., Asrul M. (2014). Effect of reagents concentration and ratio on degradation of natural rubber latex in acidic medium. Malays. J. Anal. Sci..

[23] Mariano M., Kissi N.E., Dufresne A. (2016). Cellulose nanocrystal reinforced oxidized natural rubber nanocomposites. Carbohydr. Polym..

[24] Tomić N.Z. (2020). Chapter 17—Thermal studies of compatibilized polymer blends. Compatibilization of Polymer Blends.

[25] Aini N.A.M., Othman N., Hussin M.H., Sahakaro K., Hayeemasae N. (2022). Efficiency of interaction between hybrid fillers carbon black/lignin with various rubber-based compatibilizer, epoxidized natural rubber, and liquid butadiene rubber in NR/BR composites: Mechanical, flexibility and dynamical properties. Ind. Crops Prod..

[26] Masłowski M., Miedzianowska J., Strzelec K. (2019). Silanized cereal straw as a novel, functional filler of natural rubber biocomposites. Cellulose.

[27] Hariwongsanupab N., Thanawan S., Amornsakchai T., Vallat M.F., Mougin K. (2017). Improving the mechanical properties of short pineapple leaf fiber reinforced natural rubber by blending with acrylonitrile butadiene rubber. Polym. Test..

[28] Yantaboot K., Amornsakchai T. (2017). Effect of mastication time on the low strain properties of short pineapple leaf fiber reinforced natural rubber composites. Polym. Test..

[29] Babaei-Ghazvini A., Acharya B. (2023). The effects of aspect ratio of cellulose nanocrystals on the properties of all CNC films: Tunicate and wood CNCs. Carbohydr. Polym..

[30] Chakrabarty A., Teramoto Y. (2018). Recent advances in nanocellulose composites with polymers: A guide for choosing partners and how to incorporate them. Polymers.

[31] Kazemi H., Mighri F., Park K.W., Frikha S., Rodrigue D. (2022). Natural rubber biocomposites reinforced with cellulose nanocrystals/lignin hybrid fillers. Polym. Compos..

[32] Hosseinmardi A., Amiralian N., Martin D.J., Annamalai P.K. (2024). Achieving ultra-tear resistant high-performance natural rubber nanocomposite via bio-inspired lignocellulosic compatibilization. Ind. Crops Prod..

[33] Srisuwan L., Jarukumjorn K., Suppakarn N. (2018). Effect of silane treatment methods on physical properties of rice husk flour/natural rubber composites. Adv. Mater. Sci. Eng..

[34] Maslowski M., Miedzianowska J., Strzelec K. (2019). Natural Rubber Composites Filled with Crop Residues as an Alternative to Vulcanizates with Common Fillers. Polymers.

[35] Lopattananon N., Panawarangkul K., Sahakaro K., Ellis B. (2006). Performance of pineapple leaf fiber–natural rubber composites: The effect of fiber surface treatments. J. Appl. Polym. Sci..

[36] Pickering K.L., Efendy M.G.A., Le T.M. (2016). A review of recent developments in natural fibre composites and their mechanical performance. Compos. Part A.

[37] Sainumsai W., Toki S., Amnuaypornsri S., Nimpaiboon A., Sakdapipanich J., Rong L. (2017). Dependence of the Onset of Strain-Induced Crystallization of Natural Rubber and Its Synthetic Analogue on Crosslink and Entanglement by Using Synchrotron X-ray. Rubber Chem. Technol..

[38] Cao X., Xu C., Wang Y., Liu Y., Liu Y., Chen Y. (2013). New nanocomposite materials reinforced with cellulose nanocrystals in nitrile rubber. Polym. Test..

[39] Wongsorat W., Suppakarn N., Jarukumjorn K. (2013). Effects of compatibilizer type and fiber loading on mechanical properties and cure characteristics of sisal fiber/natural rubber composites. J. Compos. Mater..

[40] Intapun J., Rungruang T., Suchat S., Cherdchim B., Hiziroglu S. (2021). The characteristics of natural rubber composites with Klason lignin as a green reinforcing filler: Thermal stability, mechanical and dynamical properties. Polymers.

[41] Bendahou A., Kaddami H., Dufresne A. (2010). Investigation on the effect of cellulosic nanoparticles’ morphology on the properties of natural rubber-based nanocomposites. Eur. Polym. J..

[42] Bokobza L. (2023). Elastomer nanocomposites: Effect of filler-matrix and filler-filler interactions. Polymers.

[43] Dayo A.Q., Gao B.C., Wang J., Liu W.B., Derradji M., Shah A.H., Babar A.A. (2017). Natural hemp fiber reinforced polybenzoxazine composites: Curing behavior, mechanical and thermal properties. Compos. Sci. Technol..

[44] Haris N.I.N., Hassan M.Z., Ilyas R., Suhot M.A., Sapuan S., Dolah R., Mohammad R., Asyraf M.R.M. (2022). Dynamic mechanical properties of natural fiber reinforced hybrid polymer composites: A review. J. Mater. Res. Technol..

[45] Low D.Y.S., Supramaniam J., Soottitantawat A., Charinpanitkul T., Tanthapanichakoon W., Tan K.W., Tang S.Y. (2021). Recent Developments in Nanocellulose-Reinforced Rubber Matrix Composites: A Review. Polymers.

[46] Peng Y., Gardner D.J., Han Y., Kiziltas A., Cai Z., Tshabalala M.A. (2013). Influence of drying method on the material properties of nanocellulose I: Thermostability and crystallinity. Cellulose.

[47] Alias N.F., Ismail H., Ishak K.M.K. (2021). Poly(lactic acid)/natural rubber/kenaf biocomposites production using poly(methyl methacrylate) and epoxidized natural rubber as co-compatibilizers. Iran. Polym. J..

[48] Vanderfleet O.M., Reid M.S., Bras J., Heux L., Godoy-Vargas J., Panga M.K.R. (2018). Insight into thermal stability of cellulose nanocrystals from new hydrolysis methods with acid blends. Cellulose.

